# Hypertrophic Gastric folds with Hypomagnesemia, linking the dots

**DOI:** 10.12669/pjms.37.4.3984

**Published:** 2021

**Authors:** Mansoor Zafar, Tila Muhammad, Najam us Saher, Muhammad Toqeer

**Affiliations:** 1 Dr. Mansoor Zafar, MBBS, MRCP (UK). Gastroenterology and General Internal Medicine Specialty Registrar, Conquest Hospital. East Sussex Healthcare, NHS Trust. TN37 7RD, UK; 2 Dr. Tila Muhammad, MBBS, FCPS (Pakistan), MRCP (UK). Consultant Gastroenterology, Conquest Hospital. East Sussex Healthcare, NHS Trust. TN37 7RD, UK; 3 Dr. Najam us Saher, Consultant and Assistant Professor, Department of Dermatology, Aga Khan University Hospital, Karachi, Pakistan. 74800; 4 Dr. Muhammad Toqeer, MBBS, FCPS (Pakistan), MRCP (UK). Consultant Gastroenterology. Conquest Hospital. East Sussex Healthcare, NHS Trust. TN37 7RD, UK

**Keywords:** Oesophago-gastroduodenoscopy (OGD), Hypertrophic Gastric Polyps, Hypomagnesemia, Zollinger-Ellison Syndrome, Multiple Endocrine Neoplasia type 1 (MEN1), Proton Pump Inhibitors (PPIs)

## Abstract

A Caucasian man in early 80s was seen in Gastroenterology Clinic, following, referral from the Endocrinology Clinic for concerns for CT Abdomen requested for tiredness and weight loss of three kilograms. The patient also had microcytic picture with low MCV and Ferritin and hypomagnesemia. The CT suggested gross circumferential thickening of the wall of stomach with advice for invasive investigations to further characterise the CT findings. The Endoscopy suggested grossly enlarged rugae in the stomach, and enlarged gastric polyps. Patient was assured no new sinister abnormality.

Treatment challenges to consider were to stop acid suppression by prescribing Proton Pump Inhibitors (PPIs) which would lead to stomach ulcers, or to continue with PPIs with sequalae of worsening of hypertrophic gastric folds, enlarged gastric polyps and hypomagnesemia. It would be necessary to consider risk versus benefits in either situation to determine an appropriate treatment plan in the long term.

With background of Zollinger-Ellison Syndrome and MEN1 with heterozygous mutation with gastrinoma of the duodenum, and frailty he was advised to continue with Proton Pump Inhibitors with twice weekly correction of Magnesium infusions, and Iron tablets following Multi-disciplinary meeting.

## CASE REPORT

A Caucasian man in early 80s was seen in Gastroenterology Clinic referred from the Endocrinology clinic for concerns over the CT-Abdomen findings, requested for complaint of tiredness, fatigue, weight loss of three kilograms, microcytic picture with low MCV and Ferritin, hypomagnesemia.

He was approached remotely by the Tertiary hospital after his father and another sibling were managed for complex renal stones, and genetic testing confirmed Multiple Endocrine Neoplasia Type-1 (MEN1) in them. Following he underwent Parathyroidectomy fifty-five years ago and again twenty-five years ago, for hypercalcemia.

He has a complex past history of MEN I, with heterozygous mutation, Zollinger-Ellison Syndrome secondary to Gastrinoma of the duodenum diagnosed more than 20 years ago with no previous abdominal surgery, bilateral adrenal adenomas asymptomatic, stable psoriasis and, hypertension. Other comorbidities include, pulmonary embolism and persistent hypomagnesaemia on twice weekly magnesium infusions, and Omeprazole 20 mg, three times a day for more than forty years, as prescribed by the tertiary centre. He was later referred to the local district general hospital for periodic follow up due to frailty. His family history with one son who had tested negative for MEN, and another child not willing to get investigated. Non-smoker, with Alcohol only socially less than 21 units/ week, lives with wife.

### Clinical Examination

Suggested normal scaphoid abdomen, with stable phase psoriatic patches along both elbows. Pulse 69, BP 170/96, Weight 66 kg (recent visit) and 69.1 kg (six months ago). It was concluded that patient needed further work-up to clarify CT findings.

### Investigations:

### Electrolytes

Serum Magnesium 0.38*, Serum Inorganic Phosphate 1.14 mmol/l, Serum Sodium 129* mmol/l, Serum Potassium 4.9 mmol/l, Corrected Calcium 2.56 mmol/l.

### Hormonal Profile

Serum FSH 21.3* IU/L, Serum LSH 7.2 IU/L, Plasma Gastrin 224* pmol/l, Plasma Vasointestinal Peptide <4, Plasma Pancreatic Polypeptide 85 pmol/l, Plasma Chromogranin B 106 pmol/l, Plasma Chromogranin A 64* pmol/l, Serum Parathormone 45 ng/l (15-65), Plasma Calcitonin <3, Plasma Glucagon 10 pmol/l, Plasma Somatostatin 23 pmol/l, Serum IGF-1 23.4, Serum Human Growth Hormone 8.64 ug/l, Serum Testosterone 25.46 nmol/l.

### Haematinics

Ferritin 33, Folate 9.2, Vitamin B12 499, MCV 79.7*, Haemoglobin 126*, Platelet Count 178.

### CT Abdomen:

**Fig.1:**
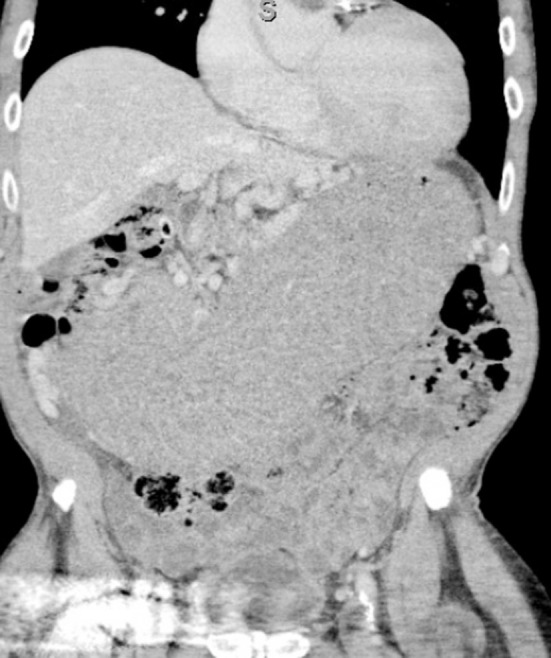
Computerised Tomogram (CT) -Abdomen; Coronal view, grossly enlarged Stomach.

**Fig.2:**
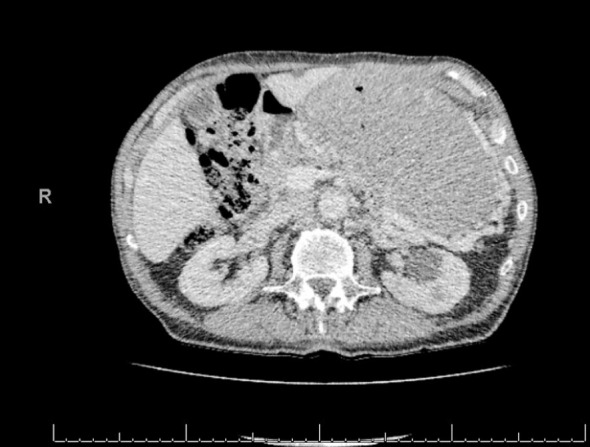
Computerised Tomogram. Transverse CT view, grossly enlarged stomach, pancreatic atrophy.

- Pancreas compressed/displaced by a grossly enlarged gastric chamber which appeared to correspond to a circumferential thickening of the wall of the stomach of uncertain aetiology.
- Subtle locules of air noted within the periphery of the gastric chamber, of uncertain significance with presumption of related to air between presumably grossly hypertrophic rugae or, much less likely, a concomitant longstanding bezoar. (Fig.1, 2).

### Oral Gastro-Duodenoscopy:

**Fig.3:**
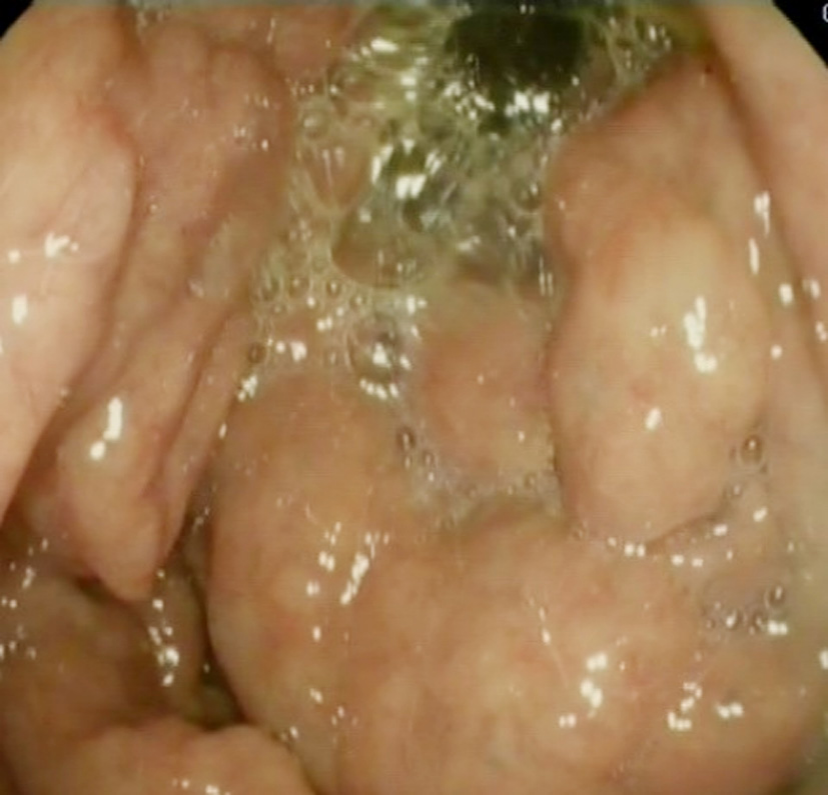
OGD; Antral view with hypertrophic Gastric Folds and Large Gastric Polyps.

**Fig.4:**
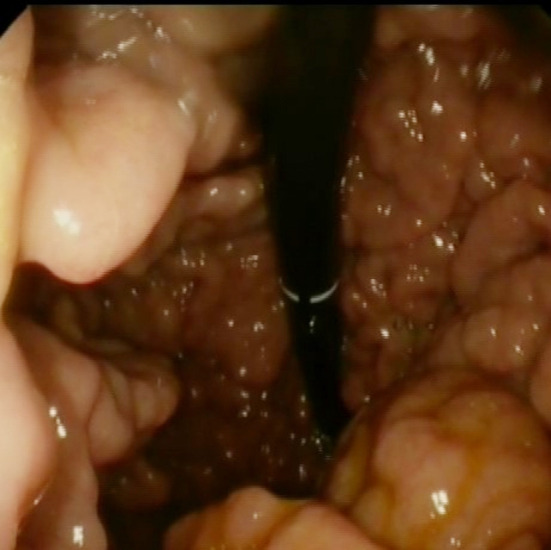
OGD; Stomach in Retro flexion. The thickening seen on the CT secondary to enlarged folds and huge fundic gland polyps secondary to chronic PPI use.

- Significant hypertrophy of gastric folds in the fundus large gastric polyps. (Fig. 3, 4).

### Treatment:

- The case was discussed in Gastroenterology Multi-Disciplinary Meeting.
- Considering the frailty, it was decided that patient need to be managed conservatively with PPIs, and periodic twice weekly 24 mmol Magnesium infusions.
- His corrected Calcium levels to date have been in normal range.

### Outcome and Follow up:

Patient was assured of no sinister pathology. He was advised periodic clinic review in Gastroenterology Clinic, with 24 mmol Magnesium infusions on regular basis twice in a week, with continuation of Omeprazole 20 mg three times a day, and Ferrous Gluconate 210 mg once daily. He reported resolution of symptoms of tiredness, and happy with the conservative management.

## DISCUSSION

Zollinger-Ellison Syndrome is neuro-endocrine functional tumours predominantly of either Pancreatic or Duodenal Origin. Incidence remains 0.2-2 per 1000000 annually.^1^ They are associated with increase Gastrin Secretion, with associated stomach ulcers, diarrhoea and other sequalae. Most patents are diagnosed in age group 20 to 50 and most of them are Men. Surprisingly 80% of cases are sporadic^2^, however, up to 30% of cases are in association with MEN1, as our patient.^3^ The Pancreatic Origin is more likely to metastasize; however, duodenal origin runs a relatively benign course, with less likelihood of being spread to liver at the time of presentation, size usually is < 1 cm and usually multiple and more likely to occur in the first part of duodenum, as in our patient.^2^,^3^ Rarely, they may arise from stomach, liver, bile duct, lymphatic channels along mesentery or pancreatic region, ovary, and even more rarely in heart, lungs-in association with small cell lung cancer.^4^

In a patient managed conservatively with PPIs on long term basis, for duodenal origin of Zollinger-Ellison Syndrome, there are multiple side-effects reported. The common side effects include: Hyponatremia, Clostridium difficile enteritis, and Interstitial Nephritis.

Komorowski et al have elaborated the importance of snare biopsy for surveillance to rule out Gastric Cancers.^5^ Kuiper EJ et al, have found more predisposition to Helicobacter pylori infection with long term use of PPIs^6^ and more incidence of Atrophic Gastritis and Gastric polyps with prolong use of PPIs.^7^

The National Institute of Diabetes and Digestive and Kidney Disease (NIDDK), gives online info for patients, general population and healthcare providers towards symptomatology, and management of the patients with Zollinger-Ellison Syndrome in particular in association with MEN1 8. National Organisation for Rare Disorders also has helpful info available.^9^

### Learning Points:

- Understanding of side-effects of long-term use of PPIs is important to clinically co-relate, with side effects.
- Endoscopic viewing is important to clarify ambiguity by the scans, to rule out stomach cancer.
- There are frail patients with Zollinger-Ellison Syndrome, associated with MEN1, that can be managed conservatively.

### Authors’ Contribution:

**MZ:** Clinically evaluated the patient, designed the case report, prepared the manuscript and is responsible for clinical integrity of the study.

**TM:** Clinically involved in the care of the patient.

**NS:** Reviewed the manuscript for intellectual input.

**MT:** Involved in the overall care of the patient, and reviewed the manuscript for intellectual input.

## References

[1] Metz DC, Jensen RT (2008). Gastrointestinal neuroendocrine tumors:pancreatic endocrine tumors. Gastroenterology.

[2] Berna MJ, Annibale B, Marignani M, Luong TV, Corleto V, Pace A (2008). A prospective study of gastric carcinoids and enterochromaffin-like cell changes in multiple endocrine neoplasia type 1 and Zollinger-Ellison syndrome:identification of risk factors. J Clin Endocrinol Metab.

[3] Norton JA (1994). Neuroendocrine tumors of the pancreas and duodenum. Curr Probl Surg.

[4] Norton JA, Alexander HR, Fraker DL, Venzon DJ, Gibril F, Jensen RT (2003). Possible primary lymph node gastrinoma:occurrence, natural history, and predictive factors:a prospective study. Ann Surg.

[5] Komorowski RA, Caya JG, Geenen JE (1986). The morphologic spectrum of large gastric folds:utility of the snare biopsy. Gastrointest Endosc.

[6] Kuipers EJ, Uyterlinde AM, Peña AS, Hazenberg HJ, Bloemena E, Lindeman J (1995). Increase of Helicobacter pylori-associated corpus gastritis during acid suppressive therapy:implications for long-term safety. Am J Gastroenterol.

[7] Kuipers EJ, Lundell L, Klinkenberg-Knol EC, Havu N, Festen HP, Liedman B (1996). Atrophic gastritis and Helicobacter pylori infection in patients with reflux esophagitis treated with omeprazole or fundoplication. N Engl J Med.

[8] Zollinger-Ellison Syndrome National Institute of Diabetes and Digestive and Kidney Disease (NIDDK).

[9] Gastritis, Giant Hypertrophic;National Organisation for Rare Disorders (NORD) [https://rarediseases.org/rare-diseases/gastritis-giant-hypertrophic/] [Years Published 1986 1994,2002 2004]

